# *N*-(2-Hydroxy­ethyl)-2-[2-(hydroxy­imino)propanamido]ethanaminium 2-(hydroxy­imino)propanoate

**DOI:** 10.1107/S1600536809029778

**Published:** 2009-08-08

**Authors:** Turganbay S. Iskenderov, Valentina A. Kalibabchuk, Irina A. Golenya, Nikolay M. Dudarenko, Natalia I. Usenko

**Affiliations:** a Karakalpakian University, Department of Chemistry, Universitet Keshesi 1, 742012 Nukus, Uzbekistan; b O. O. Bohomolets National Medical University, Department of General Chemistry, Shevchenko Blvd. 13, 01601 Kiev, Ukraine; c Kiev National Taras Shevchenko University, Department of Chemistry, Volodymyrska Str. 64, 01601 Kiev, Ukraine

## Abstract

The cation of the title salt, C_7_H_16_N_3_O_3_^+^·C_3_H_4_NO_3_^−^, the oxime group is *trans* with respect to the amide–carbonyl group. The components of the structure are united into a three-dimensional network by an extensive system of O—H⋯O and N—H⋯O hydrogen bonds.

## Related literature

For background to oximes in coordination chemistry, see: Kukushkin *et al.* (1996 ▶); Chaudhuri (2003 ▶). For polynuclear species arising from bridging and/or functionalized oximes, see: Cervera *et al.* (1997 ▶); Costes *et al.* (1998 ▶); Moroz *et al.* (2008 ▶); Onindo *et al.* (1995 ▶); Sliva *et al.* (1997*a* ▶,*b* ▶); Gumienna-Kontecka *et al.* (2000 ▶). For oximes stabilizing high oxidation states, see: Kanderal *et al.* (2005 ▶); Fritsky *et al.* (2006 ▶). For related structures, see: Duda *et al.* (1997 ▶); Fritsky *et al.* (1999 ▶); Fritsky (1999 ▶); Mokhir *et al.* (2002 ▶). For the synthesis, see: Lau & Gutsche (1978 ▶). 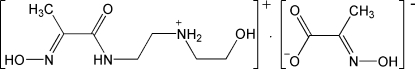


## Experimental

### 

#### Crystal data

C_7_H_16_N_3_O_3_^+^·C_3_H_4_NO_3_^−^
*M* *_r_* = 292.30
Monoclinic, 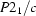

*a* = 9.355 (2) Å
*b* = 6.996 (1) Å
*c* = 20.606 (4) Å
β = 96.99 (3)°
*V* = 1338.6 (4) Å^3^
*Z* = 4
Mo *K*α radiation
μ = 0.12 mm^−1^
*T* = 120 K
0.30 × 0.24 × 0.20 mm

#### Data collection

Nonius KappaCCD diffractometer
Absorption correction: multi-scan (North *et al.*, 1968 ▶) *T*_min_ = 0.957, *T*_max_ = 0.979
8410 measured reflections
3090 independent reflections
1887 reflections with *I* > 2σ(*I*)
*R*_int_ = 0.049

#### Refinement

*R*[*F* ^2^ > 2σ(*F* ^2^)] = 0.044
*wR*(*F* ^2^) = 0.088
*S* = 0.92
3090 reflections
201 parameters
H atoms treated by a mixture of independent and constrained refinement
Δρ_max_ = 0.20 e Å^−3^
Δρ_min_ = −0.25 e Å^−3^

### 

Data collection: *COLLECT* (Bruker, 2004 ▶); cell refinement: *DENZO*/*SCALEPACK* (Otwinowski & Minor, 1997 ▶); data reduction: *DENZO*/*SCALEPACK*; program(s) used to solve structure: *SIR2004* (Burla *et al.*, 2005 ▶); program(s) used to refine structure: *SHELXL97* (Sheldrick, 2008 ▶); molecular graphics: *ORTEP-3 for Windows* (Farrugia, 1997 ▶); software used to prepare material for publication: *SHELXL97*.

## Supplementary Material

Crystal structure: contains datablocks global, I. DOI: 10.1107/S1600536809029778/tk2512sup1.cif

Structure factors: contains datablocks I. DOI: 10.1107/S1600536809029778/tk2512Isup2.hkl

Additional supplementary materials:  crystallographic information; 3D view; checkCIF report

## Figures and Tables

**Table 1:** Hydrogen-bond geometry (Å, °)

*D*—H⋯*A*	*D*—H	H⋯*A*	*D*⋯*A*	*D*—H⋯*A*
O3—H3*O*⋯O5^i^	0.90 (2)	1.78 (2)	2.677 (2)	173 (2)
O4—H4*O*⋯O2^ii^	0.95 (2)	1.63 (2)	2.5733 (18)	172.3 (19)
O6—H6*O*⋯O2	0.87 (2)	2.25 (2)	3.101 (2)	163.4 (18)
N3—H3*N*⋯O6^ii^	0.86 (2)	2.11 (2)	2.940 (2)	162.6 (18)
N4—H4*N*⋯O1^iii^	0.970 (19)	1.93 (2)	2.838 (2)	155.1 (15)
N4—H5*N*⋯O1^iv^	0.895 (18)	1.921 (19)	2.796 (2)	165.1 (17)

Symmetry codes: (i) 
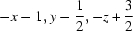
; (ii) 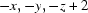; (iii) 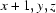; (iv) 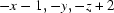.

**Fig. 1.**
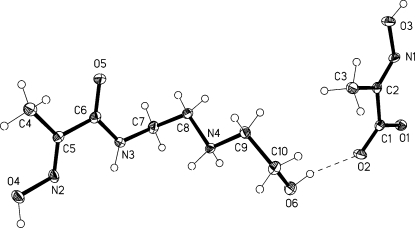
A view of compound (I), with displacement ellipsoids shown at the 50% probability level. H atoms are drawn as spheres of arbitrary radii. Hydrogen bonds are indicated by dashed lines.

## supplementary crystallographic information

### Comment

Oximes are classical type of chelating ligands traditionally widely used in coordination and analytical chemistry (Kukushkin *et al.*, 1996; Chaudhuri, 2003). They are also important bridging ligands extensively used in molecular magnetism for obtaining of polynuclear complexes (Cervera *et al.*, 1997; Costes *et al.*, 1998; Moroz *et al.*, 2008). The presence of an additional donor function in the vicinity to the oxime group may result in important increase of chelating efficiency and ability to form polynuclear species. For example, amide derivatives of 2-hydroxyiminopropanoic acid were shown to act as highly efficient chelators with respect to Cu(II), Ni(II) and Al(III) (Onindo *et al.*, 1995; Sliva *et al.*, 1997a; Sliva, *et al.*, 1997b; Gumienna-Kontecka *et al.*, 2000). Recently, owing to their strong σ-donor capacity, open-chain tetradentate oxime and amide ligands were shown to efficiently stabilize unusual oxidation states of metal ions, such as Cu^3+^ and Ni^3+^ (Kanderal *et al.*, 2005; Fritsky *et al.*, 2006). The present investigation is dedicated to the study of the molecular structure of the title compound (I), which is a new polynucleative ligand containing several donor functions: oxime, amine, amide and alcohol.

The structure of (I) is ionic and and comprises cations of *N*-[2-(2-hydroxy-ethylammonium)ethyl]-2-hydroxyiminopropanamide and 2-(hydroxyimino)propanoate anions (Fig. 1). The cation has a Γ-shaped conformation and consists of two nearly planar CH_3_C(=NOH)C(O)NHCH_2 _and CH_2_CH_2_NH_2_CH_2_ fragments. The dihedral plane between their mean planes, defined by the non-hydrogen atoms, is 75.8 (1)°. The hydroxyl group is situated nearly perpendicular to the CH_2_CH_2_NH_2_CH_2_ moiety: the torsion angle N4/C9/C10/O6 is 60.2 (2)°. The observed conformation of the CH_3_C(=NOH)C(O)NHCH_2 _moiety is the same as that observed in the structure of *N*,*N*'-bis(2-hydroxyiminopropionylpropane)-1,2-diamine and its homologues (Duda *et al.*, 1997; Fritsky, Karaczyn *et al.*, 1999). The oxime group is trans to the amide-carbonyl. It is noted that the CH_3_C(=NOH)C(O)NHCH_2 _moiety deviates somewhat from planarity because of a twisting of the oxime and amide groups along the C5—C6 bond. The dihedral angle between the corresponding least square planes is 9.5 (1)°. The C=N, C=O, N—O, C—N bond lengths are typical for 2-hydroxyiminopropanoic acid and its amide derivatives (Duda *et al.*, 1997; Fritsky, 1999; Mokhir *et al.*, 2002).

The elements of the structure are united into a 3-D network by extensive system of the O—H···O and N—H···O hydrogen bonds (Table 1).

### Experimental

Ethyl 2-(hydroxyimino)propanoate (1.31 g, 0.01 mol) was dissolved in methanol (50 ml) to which 2-((2-aminoethyl)amino)ethanol (1.04 g, 0.01 mol) was added. The mixture was set aside for 24 h at room temperature, then the solvent was removed on a rotary evaporator. Recrystallization of the crude product from water afforded the pure (I) in the form of single crystals. Ethyl 2-(hydroxyimino)propanoate was prepared according to the reported method (Lau & Gutsche, 1978).

### Refinement

The O—H and N—H hydrogen atoms were located from the difference Fourier map, and refined with *U*_iso_ = 1.5 *U*_eq_(parent atom). The remaining H atoms were positioned geometrically and were constrained to ride on their parent atoms with C—H = 0.96–0.97 Å, and with *U*_iso_ = 1.2–1.5 *U*_eq_(parent atom).

### Crystal data

**Table:**

C_7_H_16_N_3_O_3_^+^·C_3_H_4_NO_3_^−^	*F*(000) = 624
*M**_r_* = 292.30	*D*_x_ = 1.450 Mg m^−^^3^
Monoclinic, *P*2_1_/*c*	Mo *K*α radiation, λ = 0.71073 Å
Hall symbol: -P 2ybc	Cell parameters from 1078 reflections
*a* = 9.355 (2) Å	θ = 3.2–27.5°
*b* = 6.996 (1) Å	µ = 0.12 mm^−^^1^
*c* = 20.606 (4) Å	*T* = 120 K
β = 96.99 (3)°	Block, colourless
*V* = 1338.6 (4) Å^3^	0.30 × 0.24 × 0.20 mm
*Z* = 4	

### Data collection

**Table:**

Nonius KappaCCD diffractometer	3090 independent reflections
Radiation source: fine-focus sealed tube	1887 reflections with *I* > 2σ(*I*)
horizontally mounted graphite crystal	*R*_int_ = 0.049
Detector resolution: 9 pixels mm^-1^	θ_max_ = 28.4°, θ_min_ = 3.1°
φ scans and ω scans with κ offset	*h* = −12→9
Absorption correction: multi-scan (North *et al.*, 1968)	*k* = −8→9
*T*_min_ = 0.957, *T*_max_ = 0.979	*l* = −23→26
8410 measured reflections	

### Refinement

**Table:**

Refinement on *F*^2^	Primary atom site location: structure-invariant direct methods
Least-squares matrix: full	Secondary atom site location: difference Fourier map
*R*[*F*^2^ > 2σ(*F*^2^)] = 0.044	Hydrogen site location: inferred from neighbouring sites
*wR*(*F*^2^) = 0.088	H atoms treated by a mixture of independent and constrained refinement
*S* = 0.92	*w* = 1/[σ^2^(*F*_o_^2^) + (0.0375*P*)^2^] where *P* = (*F*_o_^2^ + 2*F*_c_^2^)/3
3090 reflections	(Δ/σ)_max_ = 0.002
201 parameters	Δρ_max_ = 0.20 e Å^−^^3^
0 restraints	Δρ_min_ = −0.25 e Å^−^^3^

### Special details

**Table:**

Geometry. All e.s.d.'s (except the e.s.d. in the dihedral angle between two l.s. planes) are estimated using the full covariance matrix. The cell e.s.d.'s are taken into account individually in the estimation of e.s.d.'s in distances, angles and torsion angles; correlations between e.s.d.'s in cell parameters are only used when they are defined by crystal symmetry. An approximate (isotropic) treatment of cell e.s.d.'s is used for estimating e.s.d.'s involving l.s. planes.
Refinement. Refinement of *F*^2^ against ALL reflections. The weighted *R*-factor *wR* and goodness of fit *S* are based on *F*^2^, conventional *R*-factors *R* are based on *F*, with *F* set to zero for negative *F*^2^. The threshold expression of *F*^2^ > σ(*F*^2^) is used only for calculating *R*-factors(gt) *etc*. and is not relevant to the choice of reflections for refinement. *R*-factors based on *F*^2^ are statistically about twice as large as those based on *F*, and *R*- factors based on ALL data will be even larger.

### Fractional atomic coordinates and isotropic or equivalent isotropic displacement parameters (Å^2^)

**Table:**

	*x*	*y*	*z*	*U*_iso_*/*U*_eq_	
O1	−0.84446 (13)	−0.16641 (17)	0.98706 (6)	0.0158 (3)	
O2	−0.60613 (13)	−0.22237 (19)	1.00166 (6)	0.0180 (3)	
O3	−0.84727 (14)	−0.3817 (2)	0.80265 (6)	0.0181 (3)	
H3O	−0.939 (2)	−0.373 (3)	0.7840 (10)	0.027*	
O4	0.55821 (13)	0.0801 (2)	0.88257 (6)	0.0193 (3)	
H4O	0.583 (2)	0.126 (3)	0.9258 (10)	0.029*	
O5	0.12037 (13)	0.11505 (19)	0.75400 (6)	0.0179 (3)	
O6	−0.27505 (15)	−0.2615 (2)	1.00518 (6)	0.0211 (3)	
H6O	−0.369 (2)	−0.261 (3)	0.9960 (9)	0.032*	
N1	−0.85445 (16)	−0.3060 (2)	0.86557 (7)	0.0146 (4)	
N2	0.41206 (16)	0.1204 (2)	0.87427 (7)	0.0153 (4)	
N3	0.13295 (17)	0.1750 (2)	0.86292 (8)	0.0139 (4)	
H3N	0.189 (2)	0.184 (3)	0.8990 (9)	0.021*	
N4	−0.10824 (17)	−0.0767 (2)	0.90998 (7)	0.0121 (4)	
H4N	−0.008 (2)	−0.108 (3)	0.9240 (9)	0.018*	
H5N	−0.139 (2)	−0.009 (3)	0.9426 (9)	0.018*	
C1	−0.7283 (2)	−0.2267 (3)	0.96857 (9)	0.0139 (4)	
C2	−0.73259 (19)	−0.3166 (3)	0.90150 (9)	0.0125 (4)	
C3	−0.59762 (19)	−0.4002 (3)	0.88233 (9)	0.0162 (4)	
H3A	−0.6191	−0.4704	0.8423	0.024*	
H3C	−0.5558	−0.4846	0.9162	0.024*	
H3B	−0.5309	−0.2995	0.8762	0.024*	
C4	0.4116 (2)	−0.0067 (3)	0.76127 (9)	0.0203 (5)	
H4A	0.5142	−0.0162	0.7719	0.030*	
H4B	0.3899	0.0728	0.7234	0.030*	
H4C	0.3720	−0.1319	0.7523	0.030*	
C5	0.3476 (2)	0.0790 (3)	0.81755 (9)	0.0124 (4)	
C6	0.1903 (2)	0.1255 (3)	0.80944 (9)	0.0142 (4)	
C7	−0.01883 (19)	0.2223 (3)	0.86077 (9)	0.0160 (4)	
H7A	−0.0450	0.3120	0.8254	0.019*	
H7B	−0.0345	0.2847	0.9013	0.019*	
C8	−0.1158 (2)	0.0483 (3)	0.85087 (9)	0.0143 (4)	
H8A	−0.2145	0.0903	0.8394	0.017*	
H8B	−0.0887	−0.0260	0.8145	0.017*	
C9	−0.19929 (19)	−0.2503 (3)	0.89620 (8)	0.0143 (4)	
H9A	−0.1580	−0.3293	0.8646	0.017*	
H9B	−0.2948	−0.2118	0.8770	0.017*	
C10	−0.2118 (2)	−0.3664 (3)	0.95673 (9)	0.0171 (4)	
H10A	−0.2698	−0.4789	0.9449	0.021*	
H10B	−0.1167	−0.4091	0.9750	0.021*	

### Atomic displacement parameters (Å^2^)

**Table:**

	*U*^11^	*U*^22^	*U*^33^	*U*^12^	*U*^13^	*U*^23^
O1	0.0160 (7)	0.0177 (8)	0.0139 (7)	0.0013 (6)	0.0025 (5)	−0.0025 (6)
O2	0.0156 (8)	0.0239 (8)	0.0132 (7)	0.0003 (6)	−0.0028 (6)	−0.0031 (6)
O3	0.0161 (8)	0.0264 (8)	0.0114 (7)	0.0014 (7)	−0.0003 (6)	−0.0043 (6)
O4	0.0112 (7)	0.0286 (9)	0.0177 (8)	0.0035 (6)	−0.0002 (6)	−0.0047 (6)
O5	0.0154 (7)	0.0250 (8)	0.0127 (7)	0.0001 (6)	−0.0003 (6)	0.0020 (6)
O6	0.0173 (8)	0.0311 (9)	0.0157 (7)	−0.0033 (7)	0.0057 (6)	−0.0020 (6)
N1	0.0193 (9)	0.0160 (9)	0.0089 (8)	−0.0016 (7)	0.0030 (7)	−0.0017 (7)
N2	0.0095 (8)	0.0173 (9)	0.0193 (9)	0.0018 (7)	0.0022 (7)	0.0012 (7)
N3	0.0117 (9)	0.0180 (10)	0.0116 (9)	−0.0007 (7)	−0.0001 (6)	−0.0003 (7)
N4	0.0113 (9)	0.0159 (9)	0.0094 (8)	0.0011 (7)	0.0027 (7)	−0.0001 (7)
C1	0.0160 (11)	0.0116 (11)	0.0141 (10)	−0.0007 (8)	0.0017 (8)	0.0028 (8)
C2	0.0135 (10)	0.0101 (10)	0.0139 (10)	−0.0010 (8)	0.0022 (8)	0.0014 (8)
C3	0.0142 (10)	0.0197 (11)	0.0146 (10)	0.0003 (9)	0.0010 (8)	−0.0018 (9)
C4	0.0164 (11)	0.0276 (12)	0.0167 (11)	0.0011 (9)	0.0015 (9)	−0.0019 (9)
C5	0.0152 (10)	0.0096 (10)	0.0126 (10)	−0.0005 (8)	0.0024 (8)	0.0009 (8)
C6	0.0191 (11)	0.0097 (10)	0.0138 (10)	−0.0026 (8)	0.0023 (9)	0.0030 (8)
C7	0.0151 (11)	0.0161 (11)	0.0175 (11)	0.0009 (9)	0.0042 (8)	0.0016 (8)
C8	0.0123 (10)	0.0192 (12)	0.0115 (10)	0.0014 (9)	0.0017 (8)	0.0015 (8)
C9	0.0131 (10)	0.0150 (11)	0.0150 (10)	−0.0019 (8)	0.0022 (8)	−0.0019 (8)
C10	0.0174 (11)	0.0174 (11)	0.0172 (10)	0.0015 (9)	0.0047 (8)	0.0023 (9)

### Geometric parameters (Å, °)

**Table:**

O1—C1	1.266 (2)	C2—C3	1.488 (2)
O2—C1	1.258 (2)	C3—H3A	0.9600
O3—N1	1.4095 (18)	C3—H3C	0.9600
O3—H3O	0.90 (2)	C3—H3B	0.9600
O4—N2	1.3861 (19)	C4—C5	1.494 (2)
O4—H4O	0.95 (2)	C4—H4A	0.9600
O5—C6	1.248 (2)	C4—H4B	0.9600
O6—C10	1.424 (2)	C4—H4C	0.9600
O6—H6O	0.87 (2)	C5—C6	1.497 (2)
N1—C2	1.284 (2)	C7—C8	1.518 (2)
N2—C5	1.281 (2)	C7—H7A	0.9700
N3—C6	1.329 (2)	C7—H7B	0.9700
N3—C7	1.453 (2)	C8—H8A	0.9700
N3—H3N	0.86 (2)	C8—H8B	0.9700
N4—C9	1.491 (2)	C9—C10	1.505 (2)
N4—C8	1.494 (2)	C9—H9A	0.9700
N4—H4N	0.970 (19)	C9—H9B	0.9700
N4—H5N	0.895 (18)	C10—H10A	0.9700
C1—C2	1.515 (2)	C10—H10B	0.9700
			
N1—O3—H3O	102.4 (12)	H4B—C4—H4C	109.5
N2—O4—H4O	99.8 (12)	N2—C5—C4	127.62 (17)
C10—O6—H6O	110.1 (13)	N2—C5—C6	113.55 (15)
C2—N1—O3	111.74 (14)	C4—C5—C6	118.83 (16)
C5—N2—O4	114.45 (14)	O5—C6—N3	123.72 (18)
C6—N3—C7	121.71 (16)	O5—C6—C5	119.22 (16)
C6—N3—H3N	118.4 (13)	N3—C6—C5	117.05 (16)
C7—N3—H3N	119.8 (13)	N3—C7—C8	112.78 (15)
C9—N4—C8	110.61 (14)	N3—C7—H7A	109.0
C9—N4—H4N	112.3 (11)	C8—C7—H7A	109.0
C8—N4—H4N	108.9 (11)	N3—C7—H7B	109.0
C9—N4—H5N	110.3 (12)	C8—C7—H7B	109.0
C8—N4—H5N	108.5 (12)	H7A—C7—H7B	107.8
H4N—N4—H5N	106.1 (16)	N4—C8—C7	113.05 (15)
O2—C1—O1	125.82 (17)	N4—C8—H8A	109.0
O2—C1—C2	115.20 (16)	C7—C8—H8A	109.0
O1—C1—C2	118.98 (17)	N4—C8—H8B	109.0
N1—C2—C3	126.33 (17)	C7—C8—H8B	109.0
N1—C2—C1	115.17 (16)	H8A—C8—H8B	107.8
C3—C2—C1	118.46 (16)	N4—C9—C10	112.47 (15)
C2—C3—H3A	109.5	N4—C9—H9A	109.1
C2—C3—H3C	109.5	C10—C9—H9A	109.1
H3A—C3—H3C	109.5	N4—C9—H9B	109.1
C2—C3—H3B	109.5	C10—C9—H9B	109.1
H3A—C3—H3B	109.5	H9A—C9—H9B	107.8
H3C—C3—H3B	109.5	O6—C10—C9	112.58 (15)
C5—C4—H4A	109.5	O6—C10—H10A	109.1
C5—C4—H4B	109.5	C9—C10—H10A	109.1
H4A—C4—H4B	109.5	O6—C10—H10B	109.1
C5—C4—H4C	109.5	C9—C10—H10B	109.1
H4A—C4—H4C	109.5	H10A—C10—H10B	107.8
			
O3—N1—C2—C3	−1.2 (3)	N2—C5—C6—O5	170.68 (17)
O3—N1—C2—C1	176.22 (14)	C4—C5—C6—O5	−9.1 (3)
O2—C1—C2—N1	−174.16 (17)	N2—C5—C6—N3	−9.8 (2)
O1—C1—C2—N1	7.0 (2)	C4—C5—C6—N3	170.42 (17)
O2—C1—C2—C3	3.4 (2)	C6—N3—C7—C8	73.0 (2)
O1—C1—C2—C3	−175.41 (17)	C9—N4—C8—C7	−176.88 (15)
O4—N2—C5—C4	0.4 (3)	N3—C7—C8—N4	72.22 (18)
O4—N2—C5—C6	−179.41 (14)	C8—N4—C9—C10	−171.87 (14)
C7—N3—C6—O5	−0.2 (3)	N4—C9—C10—O6	60.2 (2)
C7—N3—C6—C5	−179.75 (16)		

### Hydrogen-bond geometry (Å, °)

**Table:**

*D*—H···*A*	*D*—H	H···*A*	*D*···*A*	*D*—H···*A*
O3—H3O···O5^i^	0.90 (2)	1.78 (2)	2.677 (2)	173 (2)
O4—H4O···O2^ii^	0.95 (2)	1.63 (2)	2.5733 (18)	172.3 (19)
O6—H6O···O2	0.87 (2)	2.25 (2)	3.101 (2)	163.4 (18)
N3—H3N···O6^ii^	0.86 (2)	2.11 (2)	2.940 (2)	162.6 (18)
N4—H4N···O1^iii^	0.970 (19)	1.93 (2)	2.838 (2)	155.1 (15)
N4—H5N···O1^iv^	0.895 (18)	1.921 (19)	2.796 (2)	165.1 (17)

Symmetry codes: (i) −*x*−1, *y*−1/2, −*z*+3/2; (ii) −*x*, −*y*, −*z*+2; (iii) *x*+1, *y*, *z*; (iv) −*x*−1, −*y*, −*z*+2.
